# Creative Cognitive Reappraisal Promotes Estimation Strategy Execution in Individuals with Trait Anxiety

**DOI:** 10.3390/brainsci15040378

**Published:** 2025-04-04

**Authors:** Huan Song, Chenghui Tan, Chuanlin Zhu, Dianzhi Liu, Wenbo Peng

**Affiliations:** 1 School of Educational Science, Neijiang Normal University, Neijiang 641100, China; songh@njtc.edu.cn; 2 Key Laboratory of Applied Psychology, Chongqing Normal University, Chongqing 401331, China; 3 Department of Psychology, Renmin University of China, Beijing 100872, China; 4 School of Educational Science, Yangzhou University, Yangzhou 225002, China; 5 School of Education, Soochow University, Suzhou 225002, China

**Keywords:** trait anxiety, emotional regulation, cognitive reappraisal, creativity, estimation strategy execution

## Abstract

**Objectives:** This study aimed to investigate the impact of the creative cognitive reappraisal on the estimation strategies execution in college students with trait anxiety. **Methods:** Using the Trait Anxiety Scale, 47 participants with high (HTA) and low trait anxiety (LTA) were selected from a total of 803 college students. These participants then completed a two-digit multiplication estimation task after using cognitive reappraisal to regulate negative emotions. **Results:** The results showed that for individuals with low trait anxiety, both standard cognitive reappraisal and creative cognitive reappraisal effectively improved their negative emotional experiences, with creative cognitive reappraisal demonstrating a superior regulatory effect. For individuals with high trait anxiety, creative cognitive reappraisal was effective in regulating negative emotions, whereas the effect of standard cognitive reappraisal on emotion regulation was not significant. **Conclusions:** Both standard cognitive reappraisal and creative cognitive reappraisal can enhance the speed of estimation strategy execution in college students with trait anxiety after regulating negative emotions, with creative cognitive reappraisal showing a more pronounced facilitative effect.

## 1. Introduction

Trait anxiety refers to an individual’s tendency to evaluate internal stimuli or external events in a way that causes anxiety [1], which is characterized as a relatively enduring and stable personality trait [2]. This personality characteristic can lead individuals to automatically focus on negative information [3], resulting in individuals with high trait anxiety (HTA) habitually maintaining higher levels of anxiety [1,4], thereby influencing their cognitive processes and behavior. Recent studies have examined the execution of estimation strategies in individuals with trait anxiety. Estimation strategies are rules or procedures employed by people to achieve faster and more accurate estimation outcomes [5]. Estimation strategies are frequently used in everyday life, such as estimating the area of a place or the time needed for a journey. For instance, in multiplication estimation tasks, commonly used strategies include rounding up (RU, e.g., estimating 23 × 47 as 30 × 50), rounding down (RD, e.g., estimating 23 × 47 as 20 × 40), up-down (UD, e.g., estimating 23 × 47 as 30 × 40), and down-up (DU, e.g., estimating 23 × 47 as 20 × 50) strategies [6]. Strategy execution involves the process by which individuals apply a given strategy to solve problems based on the task at hand [7], primarily focusing on the speed and accuracy of execution [8], and serves as a robust indicator of the characteristics of an individual’s estimation strategy usage [9]. Research has found that trait anxiety impacts the execution of estimation strategies. HTA individuals exhibit poorer performance in estimation strategy execution, characterized by slower response times [10,11]. This phenomenon can be explained by the processing efficiency theory, which posits that trait anxiety consumes limited working memory resources, leaving fewer resources available for cognitive tasks [12]. Consequently, individuals experience slower execution speeds for estimation strategies due to competition for limited resources. Specifically, when a high-anxiety individual performs an estimation task, they must not only solve the task itself, but also allocate additional resources to manage anxiety. This depletes the limited working memory resources, leaving fewer available for processing the estimation task, which in turn reduces the efficiency of task performance.

Furthermore, existing research has demonstrated that negative emotions can impede the speed of estimation strategy execution [13,14]. Researchers have begun to examine whether different emotion regulation strategies can mitigate the adverse effects of negative emotional states on estimation strategies. This investigation aims to reduce the impact of such emotions on cognitive task performance. Both cognitive reappraisal and expressive suppression have been found to enhance the execution of estimation strategies in the presence of negative emotions, with cognitive reappraisal showing superior regulatory effects compared to expressive suppression [11,14]. Cognitive reappraisal involves altering one’s subjective evaluation of a situation to reframe its meaning and change the emotional response it elicits [15], thereby modulating emotional experience. For instance, the common saying, “losing money to ward off disaster”, exemplifies the use of cognitive reappraisal as a strategy to regulate emotions by reinterpreting the situation.

However, a substantial body of research has identified deficiencies in emotion regulation among individuals with trait anxiety. HTA students, for instance, are less likely to use cognitive reappraisal strategies, and exhibit abnormal usage patterns when they do employ them [16,17]. This limited and ineffective use of strategies makes it difficult for them to regulate their emotions, thereby leading to emotional dysregulation [18,19,20]. Notably, research demonstrates that trait anxious individuals can effectively regulate their emotions through the successful implementation of cognitive reappraisal strategies [21]. However, this is not an easy task for anxious individuals, as it requires a significant amount of cognitive resources [22]. As a result, researchers have actively explored effective emotion regulation strategies for individuals with trait anxiety, focusing on improving their emotion regulation abilities. Research has found that HTA individuals are less likely to use cognitive reappraisal strategies due to their weaker ability to reappraise [23]. However, when they successfully apply these strategies, their emotion regulation effectiveness becomes comparable to that of low-trait anxiety (LTA) individuals [24]. This raises the question: can different emotion regulation strategies improve the impact of negative emotions on the execution of estimation strategies in individuals with trait anxiety? A study found that HTA individuals using cognitive reappraisal to regulate negative emotions can enhance the speed of estimation strategy execution, whereas expressive suppression shows no significant improvement in this regard [11]. In other words, cognitive reappraisal is more effective for HTA individuals in mitigating the negative impact of emotions on estimation strategy execution.

Recent studies have found that creative cognitive reappraisal can more effectively regulate negative emotions induced by negative images [21,25], and HTA individuals also exhibit better regulatory effects when using creative cognitive reappraisal [18]. However, whether creative cognitive reappraisal can more efficiently mitigate the impact of negative emotions on the execution of estimation strategies in those with trait anxiety remains to be further investigated. Therefore, the present study aims to investigate the influence of creative cognitive reappraisal on the execution of estimation strategies in students with trait anxiety. This research seeks to further elucidate the crucial role of creativity in cognitive reappraisal for emotion regulation and cognitive activities in individuals with trait anxiety.

## 2. Materials and Methods

### 2.1. Participants

In October 2021, a cluster sampling method was employed at a university in Sichuan Province, with classes serving as the sampling units, and questionnaires were distributed online. The Trait Anxiety Scale was used to assess 803 undergraduate students, including 251 males and 552 females. Following previous research [26], the percentile scores of the trait anxiety scale were calculated. Students scoring in the top 27% (scale score ≥ 48) were classified into the HTA group, while those in the bottom 27% (scale score ≤ 39) were classified into the LTA group. An independent samples *t*-test revealed a significant difference between the HTA group (*M* = 52.87, *SD* = 3.73) and the LTA group (*M* = 34.72, *SD* = 4.82), with *t* (481) = −46.351, *p* < 0.001, *d* = 4.227. Subsequently, participants were invited to join the experiment on a voluntary basis from both the HTA and LTA groups. A total of 47 participants were recruited: the LTA group (13 males, 10 females) had a mean trait anxiety score of 32.52 ± 4.85, while the HTA group (12 males, 12 females) had a mean score of 54.63 ± 4.40. An independent samples *t*-test confirmed a significant difference in trait anxiety scores between the two groups, with *t* (45) = −16.37, *p* < 0.001, *d* = 4.881. The mean age of the participants was 18.63 ± 1.28 years.

To control for potential confounding effects of participants’ natural emotional states [27], the Positive and Negative Affective Scale (PANAS) was used to assess participants’ mood prior to the experiment. For positive affect (PA) scores, there was no significant difference between the LTA group (*M* = 33.57, *SD* = 6.68) and the HTA group (*M* = 30.88, *SD* = 5.60), *t* (45) = 1.45, *p* = 0.15, *d* = 0.548. Similarly, for negative affect (NA) scores, there was no significant difference between the LTA group (*M* = 21.04, *SD* = 6.56) and the HTA group (*M* = 23.50, *SD* = 6.51), *t* (45) = −1.29, *p* = 0.20, *d* = 0.385. All participants were right-handed and had normal or corrected vision. None of the participants had previously taken part in similar experiments. Upon completion of the experiment, participants received appropriate compensation. Prior to participation, all participants provided written informed consent in accordance with the 1964 Declaration of Helsinki. The experimental protocol was approved by the Ethics Committee of Chongqing Normal University (No. CANU20210420).

### 2.2. Design

Following previous studies, emotional scenario sentences were used as emotional priming stimuli [28], presenting participants with sentences describing everyday life scenarios along with given cognitive reappraisal sentences [21,29,30]. A mixed experimental design with a 2 (group: HTA vs. LTA) × 3 (type of cognitive reappraisal: creative cognitive reappraisal, standard cognitive reappraisal, free viewing) factorial structure was employed, where group membership served as a between-subjects factor and the type of cognitive reappraisal as a within-subjects factor. The dependent variables included self-reported emotion ratings, task correctness, and response times (RTs).

### 2.3. Materials

#### 2.3.1. Trait Anxiety Scale

The Trait Anxiety Subscale of the Trait Anxiety Scale (TAS) assesses individual differences in a relatively stable anxiety tendency [2]. The revised Chinese version of this subscale was used to measure participants’ trait anxiety levels [31]. It consists of 20 items rated on a 4-point Likert scale ranging from ‘not at all’ to ‘very obvious’, where higher scores indicate greater trait anxiety. The internal consistency (Cronbach’s α) of the scale was 0.88.

#### 2.3.2. Positive and Negative Affective Scale

The Positive and Negative Affective Scale (PANAS) consists of two subscales: Positive Affective (PA) and Negative Affective (NA) [27]. The PA subscale measures participants’ positive emotions, while the NA subscale measures negative emotions. Each subscale contains 10 items, and each item is rated on a 5-point Likert scale. In this study, the internal consistency (Cronbach’s α) of the PA and NA subscales was 0.84 and 0.86, respectively.

#### 2.3.3. Multiplication Estimation Problems

Based on previous studies [13,32], the selection of multiplication estimation problems followed these principles: (1) the tens digit of all multipliers was not 0 or 5, excluding equations such as 21 × 30, 25 × 37, 20 × 40, and 25 × 45; (2) the tens digit of all multipliers was not 1 or 9, excluding equations such as 13 × 38, 42 × 98, and 12 × 98; (3) the tens digits of all multipliers were different, excluding equations such as 51 × 57; (4) the tens and units digits of any multiplier were not the same, excluding equations such as 22 × 47 and 31 × 55; (5) equations with identical multipliers were excluded, such as 34 × 34 and 46 × 46; (6) equations where the rounded multipliers using the DU strategy were the same in two consecutive trials were excluded, such as 26 × 42 and 27 × 54; (7) prior research has found a multiplier order effect among Chinese participants [33], where performance improves when the first number in the multiplication problem is smaller than the second (e.g., 35 × 37 is easier than 37 × 35). Therefore, all estimation problems were designed such that the first multiplier is smaller than the second (e.g., 17 × 54), excluding reverse pairs like 54 × 17. Following these criteria, 60 estimation problems suitable for the DU strategy were selected.

#### 2.3.4. Cognitive Reappraisal Sentences

A total of 150 negative emotional scenario sentences and 150 corresponding creative cognitive reappraisal sentences were selected from the College Student Creative Cognitive Reappraisal Sentence Database [34]. For example, a negative emotional scenario (e.g., “Someone crossed my boundaries”) was paired with standard (“Endure temporarily to calm the storm and step back to broaden the horizon”) and creative (“This is an opportunity to exercise mind and wisdom”) cognitive reappraisals. Each negative emotional scenario sentence was paired with one corresponding creative cognitive reappraisal sentence. The average valence of the negative emotional scenario sentences was 2.63 ± 0.29, arousal was 6.83 ± 0.30, and dominance was 3.90 ± 0.35. These sentences successfully elicited typical negative emotions characterized by low valence (negative affect), high arousal (intense physiological activation), and low dominance (sense of losing control). These scenario sentences ranged in length from 9 to 18 Chinese characters. To ensure suitability and effectiveness ratings greater than 5, we selected creative cognitive reappraisal sentences with higher creativity scores for each corresponding negative emotional scenario. The creativity scores of the creative cognitive reappraisal sentences ranged from 5.21 to 7.29, and these sentences ranged in length from 10 to 18 Chinese characters. The mean scores (*M* ± *SD*) for creativity, suitability, and effectiveness of the creative cognitive reappraisal sentences were 6.12 ± 0.41, 6.02 ± 0.42, and 6.16 ± 0.43, respectively.

To obtain standard cognitive reappraisal sentences, which have lower levels of creativity, researchers created these sentences based on the eight most commonly used cognitive reappraisal strategies, such as generating a sentence like, “This is special effects makeup”, using the reality-checking strategy [35]. The length and complexity of the standard cognitive reappraisal sentences were similar to those of the creative cognitive reappraisal sentences, ranging from 9 to 16 Chinese characters. Ultimately, each of the 150 emotional scenario sentences was paired with two types of cognitive reappraisal sentences: creative cognitive reappraisal and standard cognitive reappraisal. In September 2021, thirty participants interested in the experiment were recruited to rate the creative cognitive reappraisal and standard cognitive reappraisal sentences on three dimensions—creativity, effectiveness, and appropriateness—using a 9-point scale (1 = almost none, 9 = very high). Before the evaluation began, the researchers explained the experimental procedure and requirements to ensure that participants fully understood the meanings of creativity, appropriateness, and effectiveness. Participants were instructed to use the 9-point scale to rate the creative cognitive reappraisal sentences on these three dimensions. Participants were then presented with practice items to familiarize themselves with the task. To balance order effects, three different experimental sequences were used for the assessments: (1) Effectiveness–Creativity–Appropriateness; (2) Creativity–Appropriateness–Effectiveness; and (3) Appropriateness–Effectiveness–Creativity. Participants were randomly and evenly assigned to one of the three sequences. Assessment orders were pseudo-randomized and balanced across participants. The entire evaluation process was divided into three phases, with each phase focusing on one of the three dimensions (creativity, appropriateness, or effectiveness). Each phase included the random presentation of 150 emotional scenario sentences and their corresponding 300 cognitive reappraisal sentences (approximately 1 to 2 h per phase). When a target emotional scenario sentence appeared on the screen, both the corresponding standard cognitive reappraisal sentence and the creative cognitive reappraisal sentence were presented simultaneously. This design allowed for direct comparison between the two types of cognitive reappraisal sentences, enhancing the evaluation’s effectiveness. After the assessment, debriefing sessions were conducted with the participants to mitigate any unpleasant feelings caused by the negative emotional scenario sentences.

For the formal experiment, 66 emotional scenario sentences and their corresponding two types of cognitive reappraisal sentences (creative cognitive reappraisal and standard cognitive reappraisal) were selected from the initial pool of 150 items, resulting in 66 creative cognitive reappraisal sentences and 66 standard cognitive reappraisal sentences. Six of these items were used as practice materials. Ultimately, each of the 66 emotional scenario sentences was paired with both types of cognitive reappraisal sentences. During the free viewing condition, participants freely read the emotional scenario sentences and responded naturally. The average length of the emotional scenario sentences was 13.22 ± 1.59 Chinese characters, the standard cognitive reappraisal sentences were 13.10 ± 1.09 characters, and the creative cognitive reappraisal sentences were 13.35 ± 0.92 characters. A one-way ANOVA revealed no significant difference in sentence length, *F* (2, 177) = 0.62, *p* > 0.05. For the standard cognitive reappraisal sentences, creativity scores ranged from 3.80 to 5.43, effectiveness scores from 5.10 to 6.80, and appropriateness scores from 5.33 to 7.00. In contrast, the creative cognitive reappraisal sentences yielded creativity scores of 6.03 to 7.27, effectiveness scores of 5.20 to 7.03, and appropriateness scores of 5.00 to 7.27, all measured on a 9-point Likert scale. A one-way ANOVA revealed significant differences between creative cognitive reappraisal and standard cognitive reappraisal in terms of creativity, *F* (1, 118) = 1009.25, *p* < 0.001, and effectiveness, *F* (1, 118) = 7.63, *p* < 0.01, but not in terms of appropriateness, *F* (1, 118) = 3.49, *p* > 0.05 (Table 1).

### 2.4. Procedure

The experiment was conducted using E-Prime version 3.0. Participants were seated at a distance of approximately 70 cm from a 21-inch CRT computer screen with a refresh rate of 100 Hz.

The experiment consisted of one block with a total of 60 trials. The 60 emotional scenario sentences were randomly assigned to three conditions for each participant, with 20 trials per condition. One-way ANOVA revealed no significant differences in text length (*F* (2, 57) = 0.88, *p* > 0.05), valence (*F* (2, 57) = 0.02, *p* > 0.05), or arousal (*F* (2, 57) = 0.57, *p* > 0.05) between the three groups. Among the 60 emotional scenario sentences, each participant was presented with 20 sentences for each of the three types of cognitive reappraisal: creative cognitive reappraisal, standard cognitive reappraisal, and free viewing. To balance order effects, three different experimental sequences were used based on the type of cognitive reappraisal. The 47 participants were randomly and evenly assigned to one of the three experimental sequences. Each participant only saw one type of cognitive reappraisal for each given emotional scenario sentence, thereby avoiding potential mutual influences between different types of cognitive reappraisal sentences for the same scenario.

**Figure 1:**
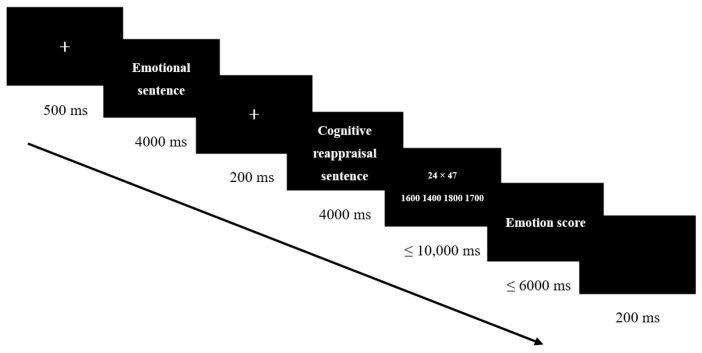
Flowchart of the study.

In the formal experiment, each trial proceeded as follows: Initially, a fixation point was presented for 500 ms, followed by an emotional scenario sentence displayed for 4000 ms. Participants were instructed to read the sentence at a natural pace and carefully understand its meaning. Subsequently, a white fixation point “+” appeared for 200 ms. Next, a cognitive reappraisal sentence (either creative or standard) was shown for 4000 ms, during which participants were asked to read it at a natural pace and use it to regulate their emotions accordingly. Following the cognitive reappraisal sentence, a multiplication estimation task appeared in the upper middle of the screen, with four multiple-choice answers aligned horizontally in the lower middle of the screen. Participants were required to select the correct answer using the DU strategy by pressing the keys “D,” “F,” “J,” and “K” in a left-to-right sequence. The position of the correct answer was counterbalanced across trials, ensuring an equal probability of appearing in any of the four positions. Participants were instructed to complete the estimation task as quickly and accurately as possible, and could respond immediately upon the appearance of the problem. If no response was made within 10,000 ms, the trial automatically advanced to the next screen. To balance position effects, the correct answer had an equal probability of appearing in any of the four positions. Immediately after the estimation task, participants were presented with an emotion rating task for 6000 ms. They were asked to rate their current emotional state on a 9-point scale (1 = not at all, 5 = moderate, 9 = extremely intense) using the numeric keys. If no response was made within 6 s, the trial was marked as invalid, and the experiment advanced to the next trial. Prior to the start of the subsequent trial, the screen remained blank for 200 ms. Throughout the experiment, text was presented in white font on a standard black background. The font size for the estimation task was 58 points, while the font size for multiple-choice answers and emotion ratings was 24 points. All elements, including the fixation point, emotional scenario sentences, cognitive reappraisal sentences, multiplication estimation problems, multiple-choice answers, and emotion ratings, were centered on the screen. The experimental procedure is illustrated in Figure 1.

## 3. Results

### 3.1. Subjective Emotional Ratings for College Students with HTA and LTA

A two-way repeated-measures ANOVA was conducted on the subjective emotional ratings of 57 participants, using group (HTA vs. LTA) and emotion regulation strategy (creative cognitive reappraisal, standard cognitive reappraisal, free viewing) as within-subjects factors. Descriptive statistics are presented in Table 2.

**Figure 2:**
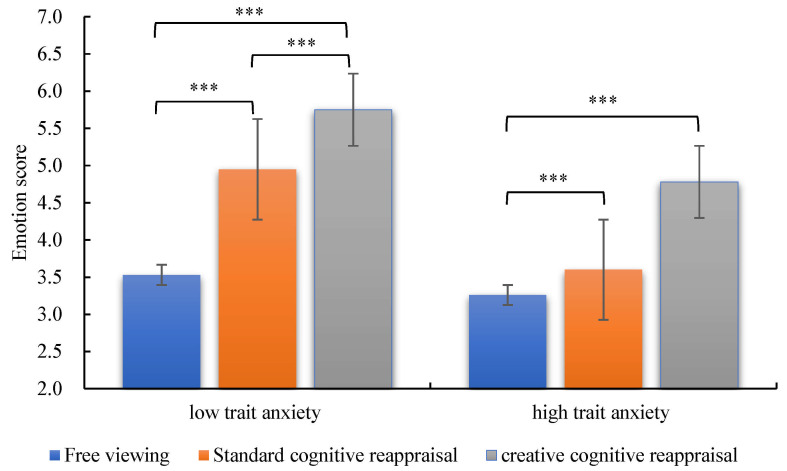
The participant emotional ratings in the LTA and HTA groups after using free viewing, standard cognitive reappraisal, and creative cognitive reappraisal to regulate negative emotions. Error bars represent standard error of the mean. *** *p* < 0.001.

The main effect of emotion regulation strategy was significant, *F* (2, 90) = 85.90, *p* < 0.001, η_p_^2^ = 0.656. Post hoc comparisons revealed that the emotional ratings under the free viewing condition (3.40 ± 0.17) were significantly lower than those for both standard cognitive reappraisal (4.28 ± 0.19, *p* < 0.001) and creative cognitive reappraisal (5.26 ± 0.16, *p* < 0.001). Additionally, the emotional ratings for creative cognitive reappraisal were significantly higher than those for standard cognitive reappraisal (*p* < 0.001). The main effect of group was also significant, *F* (1, 45) = 7.81, *p* < 0.01, η_p_^2^ = 0.148, indicating that the LTA group (4.74 ± 0.22) had significantly higher subjective emotional ratings compared to the high trait anxiety group (3.88 ± 0.22, *p* < 0.01). In addition, there was a significant interaction between emotion regulation strategy and group, *F* (2, 90) = 7.36, *p* < 0.001, η_p_^2^ = 0.141 (see Figure 2).

Further simple effects analysis indicated that for LTA individuals, the subjective emotional ratings for creative cognitive reappraisal were significantly higher than those for standard cognitive reappraisal and free viewing (*p* < 0.001). Additionally, the subjective emotional ratings for standard cognitive reappraisal were significantly higher than those for free viewing (*p* < 0.001). For HTA individuals, the subjective emotional ratings for creative cognitive reappraisal were also significantly higher than those for both standard cognitive reappraisal and free viewing (*p* < 0.001). However, there was no significant difference in subjective emotional ratings between standard cognitive reappraisal and free viewing (*p* = 0.22).

### 3.2. Accuracy and Reaction Times of Estimation Strategy Execution for HTA and LTA Individuals

A 2 (group: HTA, LTA) × 3 (type of cognitive reappraisal: creative cognitive reappraisal, standard cognitive reappraisal, free viewing) repeated-measures ANOVA was conducted to analyze the accuracy and reaction times of estimation strategy execution. Descriptive statistics are presented in Table 3.

**Figure 3:**
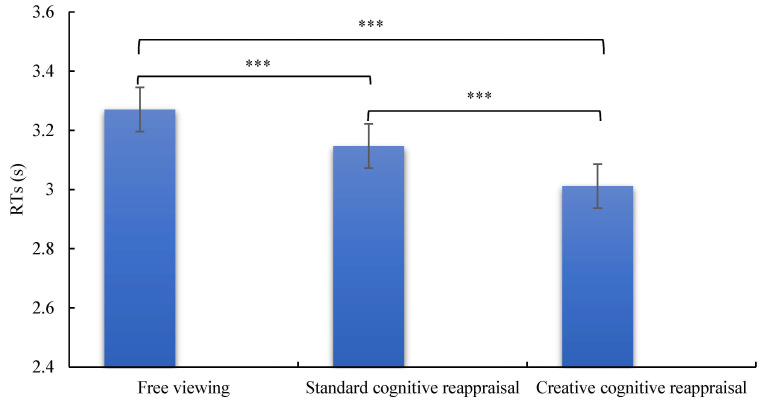
Reaction time (RT) comparison between LTA and HTA groups during an estimation task following emotion regulation via free viewing, standard cognitive reappraisal, and creative cognitive reappraisal. *** *p* < 0.001.

For the accuracy of estimation strategy execution, neither the main effects of type of cognitive reappraisal nor group, nor their interaction, reached statistical significance (*p* > 0.05). For reaction times, the main effect of type of cognitive reappraisal was significant, *F* (2, 90) = 15.38, *p* < 0.001, η_p_^2^ = 0.255, (see Figure 3). Post hoc comparisons revealed that the reaction time for the free viewing condition (3311.36 ± 878.35) was significantly longer than those for both standard cognitive reappraisal (3184.21 ± 844.07, *p* < 0.05) and creative cognitive reappraisal (3049.94 ± 831.61, *p* < 0.001). Additionally, the reaction time for standard cognitive reappraisal was found to be significantly longer than that for creative cognitive reappraisal (*p* < 0.05). The main effect of group on reaction times was not significant, *F* (1, 45) = 0.09, *p* = 0.75, η_p_^2^ = 0.002, indicating no significant difference in reaction times between the LTA group and the high trait anxiety group (*p* < 0.001). Furthermore, the interaction effect between type of cognitive reappraisal and group on reaction times was also not significant, *F* (2, 90) = 1.79, *p* = 0.17, η_p_^2^ = 0.038.

## 4. Discussion

Previous research has found that college students with HTA and LTA can improve the speed of their estimation strategy execution after using cognitive reappraisal to regulate negative emotions [11]. Therefore, this study further investigates whether the use of creative cognitive reappraisal to modulate negative emotions can more effectively enhance the execution of estimation strategies in individuals with trait anxiety. Given that individuals find it challenging to spontaneously generate creative cognitive reappraisals [1], the current study utilized emotional scenarios from the daily lives of college students as materials. By presenting given methods of cognitive reappraisal, this study examined whether using creative cognitive reappraisal to regulate negative emotions could more efficiently promote the execution of estimation strategies in individuals with trait anxiety.

### 4.1. Advantage Effect of Creative Cognitive Reappraisal in Regulating Negative Emotions

The study findings indicate that the main effects of the creativity of cognitive reappraisal and group, as well as their interaction, were all significant regarding subjective emotional ratings. First, both creative cognitive reappraisal and standard cognitive reappraisal effectively regulated negative emotions, with creative cognitive reappraisal demonstrating a superior effect. This indicates that the higher the level of creativity in cognitive reappraisal, the stronger its capacity to regulate emotions, which aligns with previous research findings [11,18]. Second, the main effect of group was significant, showing that compared to LTA students, those with HTA exhibited weaker abilities to regulate negative emotions using both creative and standard cognitive reappraisal. This finding contrasts with earlier studies [18], possibly due to the use of negative emotional scenarios from the daily lives of college students in this study, which may more effectively evoke negative emotions in individuals with trait anxiety, thereby highlighting the differences. Moreover, the interaction between the creativity of cognitive reappraisal and group was also significant. For LTA students, both standard and creative cognitive reappraisal methods were effective in regulating negative emotions, with creative cognitive reappraisal showing a better regulation effect. In contrast, for HTA students, only creative cognitive reappraisal was effective in regulating negative emotions, while the effect of standard cognitive reappraisal was not significant. This indicates that providing given sentences for creative cognitive reappraisal can assist students with trait anxiety in better regulating their negative emotions [18]. However, standard or less creative forms of cognitive reappraisal are insufficient to effectively regulate negative emotions in HTA students.

Previous studies have found that HTA individuals tend to use cognitive reappraisal strategies less frequently in daily life [16,24], and exhibit abnormal patterns in the use of these strategies [17]. Therefore, it is important to explore ways to increase the frequency of cognitive reappraisal use and enhance the creativity of cognitive reappraisal among college students with trait anxiety. Research has shown that cognitive reappraisal training can improve an individual’s ability to regulate negative emotions [36,37]. Given these findings, it may be beneficial to provide college students with trait anxiety with training in creative cognitive reappraisal to enhance their cognitive reappraisal skills and help them better regulate negative emotions. However, whether such training can effectively improve the cognitive reappraisal abilities and creativity of college students with trait anxiety remains to be further investigated. To address this, future research could design experimental or intervention studies that include systematic training programs in creative cognitive reappraisal. Creative cognitive reappraisal training can be implemented in educational settings through two phases: theoretical foundation and creative transfer. During the theoretical foundation phase, this strategy can be integrated into mental health curricula for college students by aligning it with existing course objectives. Students can be taught to apply creative cognitive reappraisal techniques to systematically analyze and reinterpret emotional experiences, thereby enhancing their emotional regulation skills. In the creative transfer phase, practical implementation can occur through peer support groups or virtual reality (VR) scenario simulations, which provide immersive, real-world contexts for skill application. Future research should focus on evaluating the efficacy of curriculum integration and peer-mediated interventions in developing creative cognitive reappraisal training programs, with a particular emphasis on longitudinal outcomes and scalability across diverse educational environments.

### 4.2. Cognitive Reassessment of Creativity as a Facilitator of Estimation Strategy Execution in Students with Trait Anxiety

Studies on estimation strategies among college students have found that accuracy is not a sensitive indicator of estimation strategy execution [11,14]. Our study observed that negative emotions did not affect the accuracy of estimation strategy execution in students with trait anxiety. A possible explanation for this finding is that the participants were undergraduate students, and for them, the multiplication estimation task was relatively simple, requiring fewer cognitive resources. According to the cognitive resource limitation theory, an individual’s cognitive resources are limited; although cognitive reappraisal consumes some of these resources, the estimation task in this study was sufficiently easy that it required minimal cognitive resources [38]. As a result, individuals with trait anxiety had enough cognitive resources to complete the task successfully, leading to an insensitivity in the accuracy measure.

In terms of reaction times, the main effect of the creativity of cognitive reappraisal was significant, indicating that the level of creativity in cognitive reappraisal influences the speed at which college students with trait anxiety complete estimation tasks. This study demonstrates that both creative cognitive reappraisal and standard cognitive reappraisal can enhance the speed of estimation task completion after regulating negative emotions in students with trait anxiety. However, creative cognitive reappraisal exhibits a more pronounced facilitative effect. This indicates that the higher the level of creativity in cognitive reappraisal, the more effectively it can mitigate the negative impact of negative emotions on the execution of estimation strategies in students with trait anxiety. In other words, increasing the creativity of cognitive reappraisal can lead to better regulation of negative emotions, thereby promoting faster and more efficient task performance among these individuals. These findings underscore the importance of incorporating highly creative elements into cognitive reappraisal strategies to improve the efficiency of cognitive processes in students with trait anxiety, particularly in the context of task performance under emotional influence. The observed effect size for reaction time improvements indicates moderate practical significance, suggesting that enhanced cognitive processing speed and working memory efficiency may positively correlate with academic performance. Specifically, faster reaction times could optimize task execution in time-sensitive academic tasks, such as problem-solving or reading comprehension. Future investigations could employ longitudinal studies with neuroimaging techniques to assess whether sustained improvements in reaction time predict specific academic outcomes (e.g., fluid intelligence, mathematical achievement) and elucidate the neurocognitive mechanisms underlying this relationship.

Additionally, the current study still has some limitations. First, this study only selected one type of estimation strategy, and whether the research results can be generalized to other types of estimation strategies or cognitive tasks requires further study. Future research can incorporate other estimation strategies, such as verbal problem-solving. Second, this study used given creative cognitive reappraisal sentences, which, although they can verify that high-creativity cognitive reappraisal can better regulate negative emotions in students with trait anxiety and promote their estimation strategy execution, it is because people with trait anxiety find it difficult to spontaneously generate highly creative cognitive reappraisals. Therefore, future research can explore how to enhance the creative cognitive reappraisal abilities of students with trait anxiety. Finally, this study has a notable limitation in its failure to distinguish among subtypes of negative emotions. Future research should investigate how creative cognitive reappraisal differentially impacts these emotion categories and their influence on estimation strategy execution in trait anxious college students.

## 5. Conclusions

In summary, our study found that both standard cognitive reappraisal and creative cognitive reappraisal can effectively improve the negative emotional experiences of college LTA students, with creative cognitive reappraisal yielding better results. For college HTA students, using creative cognitive reappraisal can effectively regulate negative emotions, whereas the effect of standard cognitive reappraisal on emotion regulation is not significant. Compared to free viewing, using either standard cognitive reappraisal or creative cognitive reappraisal to regulate negative emotions can enhance the speed of estimation strategy execution in college students with trait anxiety, and the facilitative effect of creative cognitive reappraisal is superior.

## Figures and Tables

**Table 1:** Differences in creativity, effectiveness, and appropriateness scores between standard and creative cognitive reappraisal (*M* ± *SD*).

Group	Creativity	Effectiveness	Appropriateness
Standard Cognitive Reappraisal	4.40 ± 0.36	5.97 ± 0.45	6.35 ± 0.43
Creative Cognitive Reappraisal	6.41 ± 0.33	6.20 ± 0.45	6.20 ± 0.001
*F*	1009.25 ***	7.63 **	3.49

** *p* < 0.01, *** *p* < 0.001.

**Table 2:** Subjective emotional ratings (*M* ± *SD*) with different levels of trait anxiety.

Group	Creative Cognitive Reappraisal	Standard Cognitive Reappraisal	Free Viewing
LTA	5.75 ± 1.18	4.95 ± 1.51	3.53 ± 1.45
HTA	4.78 ± 1.08	3.60 ± 1.08	3.26 ± 0.76

**Table 3:** Accuracy and reaction times (*M* ± *SD*) for estimation strategy execution with different levels of trait anxiety.

Group	Emotion Regulation Strategy	Accuracy (%)	Reaction Time (ms)
LTA	Free Viewing	98.04 ± 2.00	3361.44 ± 760.39
	Standard Cognitive Reappraisal	97.61 ± 2.32	3165.96 ± 766.55
	Creative Cognitive Reappraisal	97.83 ± 2.34	3115.34 ± 749.12
HTA	Free Viewing	94.40 ± 1.92	3261.28 ± 831.31
	Standard Cognitive Reappraisal	94.20 ± 2.22	3202.48 ± 766.06
	Creative Cognitive Reappraisal	94.40 ± 2.25	2984.55 ± 749.66

## Data Availability

The dataset will be made available upon request, in accordance with the prevailing privacy and ethical restrictions.
